# Reform of the Poor Law

**Published:** 1921-02-12

**Authors:** 


### Reform of the Poor Law.

What is generally understood by the reform of the Poor Law?—a subject which is just now being energetically discussed in this country by sociologists, members of health organisations, and by a good many persons whose duty it is to administer the Poor Law. To most people, we believe, it means the abolition of the main features of the present system; but we have never yet been able to ascertain exactly what is proposed as a substitute. That there must exist some system of the kind, call it by what name you will, is obvious. The poor are always with us, and the many problems which their presence creates must be dealt with on some such basis as has been evolved through a long process of careful legislation. After all, among all our local Government institutions, the Poor Law, as the Countess of Selborne pointed out some time ago in the *Woman's Leader*, is in the most direct contact with the democracy. The administration of each small area is under the direct supervision of a popularly elected body; and over it is the restraining hand of the Ministry of Health to curb those sentimental excesses of relief work which constitute one of the greatest dangers of the present day, for they not only cause a serious drain upon the rates, but are calculated to lower the self-respect of a large element in the community, and therefore to add steadily to the number of those who are too willing to receive public assistance.

Clearly we cannot abolish workhouses while there are the destitute to be maintained; nor can we dispense with the relieving officers, whose duties are the very backbone of the system. What is really needed is not that the system should be scrapped but that it should be extended and developed. There ought to be closer co-operation between county and district, and a unification of the medical service. We also agree that casuals ought to be transferred to the care of the police, a change the reason for which is already implicitly recognised by the Ministry in the fact that the Registrar-General has placed upon the police the responsibility of counting the tramps in the coming Census. The right place for the casual ward is at the police station, and the expense of maintenance should be put upon the Home Office, for guardians cannot give effective supervision to this form of relief, and it is not fair to put it on the local rates, as vagrants are homeless. Moreover, as the Countess of Selborne pointed out, the whole apparatus of forced labour and compulsory detention brings in a spirit entirely alien to the sympathetic assistance of misfortune which is the *raison d'être* of the rest of the Poor-law machinery. If, instead of its abolition, the Poor Law were converted into a far more comprehensive machine, so as to be the means by which all relief of distress out of public funds is distributed, a great deal of administrative inefficiency would be prevented and real economies would be effected. Pensions should come within this category, as well as, it is suggested, the granting of milk to nursing and expectant mothers. We believe that in any readjustment of the Poor-law system the two main difficulties that the reformers will have to overcome will be, on the one hand, to avoid the pitfalls of sentimental socialism, and, on the other, to clear the workhouse of the workhouse taint; to modify the spirit of the Poor Law so that it may help an unfortunate man without "pauperising" him. We have not yet satisfactorily solved the problem of the workhouse child, and, until we do, the workhouse child of to-day will be the adult and confirmed pauper of to-morrow.

